# In Search of an Efficient Complexing Agent for Oxalates and Phosphates: A Quantum Chemical Study

**DOI:** 10.3390/nano11071763

**Published:** 2021-07-06

**Authors:** Jelle Vekeman, Javier Torres, Cristina Eugenia David, Els Van de Perre, Karl Martin Wissing, Emmanuel Letavernier, Dominique Bazin, Michel Daudon, Agnieszka Pozdzik, Frederik Tielens

**Affiliations:** 1 General Chemistry (ALGC), Materials Modelling Group, Vrije Universiteit Brussels, 1050 Brussels, Belgium; 2 Grupo de Química Computacional y Teórica (QCT-USFQ), Departamento de Ingeniería Química, Universidad San Francisco de Quito (USFQ), Diego de Robles y Vía Interoceánica, Quito 17-1200-841, Ecuador; 3 Instituto de Simulación Computacional (ISC-USFQ), Departamento de Ingeniería Química, Universidad San Francisco de Quito (USFQ), Diego de Robles y Vía Interoceánica, Quito 17-1200-841, Ecuador; 4 Kidney Stone Clinic, Nephrology Department, Centre Hospitalier Universitaire, Brugmann Hospital, 1020 Brussels, Belgium; CristinaEugenia.DAVID@chu-brugmann.be (C.E.D.); Agnieszka.POZDZIK@chu-brugmann.be (A.P.); 5 Faculty of Medicine, Université Libre de Bruxelles (ULB), 1050 Brussels, Belgium; 6 Nephrology Department, Universitair Ziekenhuis Brussel, Vrije Universiteit Brussel, 1090 Brussels, Belgium; Els.VandePerre@uzbrussel.be (E.V.d.P.); KarlMartin.Wissing@uzbrussel.be (K.M.W.); 7 Sorbonne Universités-UPMC Univ. Paris 06, UMR S 1155, 75020 Paris, France; emmanuel.letavernier@aphp.fr (E.L.); michel.daudon@aphp.fr (M.D.); 8 INSERM, UMR S 1155, 75020 Paris, France; 9 Explorations Fonctionnelles Multidisciplinaires, AP-HP, Hôpital Tenon, 75020 Paris, France; 10 Institut de Chimie Physique, UMR CNRS 8000, Université Paris Saclay, Bâtiment 350, CEDEX, 91405 Orsay, France; bazin@lps.u-psud.fr; 11 Laboratoire de Physique des Solides, UMR CNRS 8502, Université Paris-Saclay, Bâtiment 510, CEDEX, 91405 Orsay, France

**Keywords:** oxalates, phosphates, complexation, nephrology, DFT

## Abstract

Limiting gastrointestinal oxalate absorption is a promising approach to reduce urinary oxalate excretion in patients with idiopathic and enteric hyperoxaluria. Phosphate binders, that inhibit gastrointestinal absorption of dietary phosphate by the formation of easily excretable insoluble complexes, are commonly used as a treatment for hyperphosphatemia in patients with end-stage renal disease. Several of these commercially available phosphate binders also have affinity for oxalate. In this work, a series of metallic cations (Li^+^, Na^+^, Mg^2+^, Ca^2+^, Fe^2+^, Cu^2+^, Zn^2+^, Al^3+^, Fe^3+^ and La^3+^) is investigated on their binding affinity to phosphate and oxalate on one side and anionic species that could be used to administer the cationic species to the body on the other, e.g., acetate, carbonate, chloride, citrate, formate, hydroxide and sulphate. Through quantum chemical calculations, the aim is to understand the competition between the different complexes and propose possible new and more efficient phosphate and oxalate binders.

## 1. Introduction

Kidney stone (KS) formation is a very frequent condition, with an estimated prevalence ranging from 9% to 13% in males and from 5.8% to 7.8% in females in western countries [1,2,3]. On top of that, recurrent stone formation occurs in up to 50% of patients [4,5,6]. KS formation hence constitutes a significant source of morbidity and treatment-related costs [7]. From a physicochemical point of view, KSs are composed of both organic and inorganic crystals organized in aggregations of micrometre scale crystallites formed inside the renal tubular lumina or the urinary tract [7,8]. A morphoconstitutional classification categorizes KS in 7 classes and 22 subclasses which correlate with different causative pathologies [4,8]. The major component of more than two-thirds of all KS is calcium oxalate in the form of calcium oxalate monohydrate and calcium oxalate dihydrate [9,10,11].

Urinary oxalate excretion is an important determinant for the formation of calcium oxalate KSs. Urinary oxalate originates from endogenous production, as the end-product of amino acid metabolism and from gastrointestinal absorption of dietary oxalate, which can account for up to 50% of urinary oxalate in healthy individuals [12]. Hyperoxaluria, defined as urinary oxalate excretion higher than 45 mg/24 h, can be categorized into three forms: (1) rare hereditary disorders of the hepatic glyoxylate metabolism leading to increased endogenous oxalate production and (2) enteric hyperoxaluria, i.e., increased gastro-intestinal oxalate absorption due to malabsorption. The most common type of hyperoxaluria, however, is (3) idiopathic hyperoxaluria, caused by high dietary oxalate intake and probably additional gastrointestinal oxalate hyperabsorption [13].

Thus, limiting gastrointestinal oxalate absorption is a valuable approach for the reduction of urinary oxalate excretion in idiopathic and enteric hyperoxaluria. Unfortunately, current therapeutic options are limited and comprise increased dietary calcium intake, possibly combined with calcium supplementation [14,15,16]. Furthermore, this intake is needed to avoid hyperphosphatemia, which is common in chronic kidney disease and is associated with cardiovascular morbidity and mortality [17].

From the 1970s on, three different types of phosphate binders have been used during overlapping timespans as a treatment for hyperphosphatemia: alkaline aluminium salts were used until the toxicity of aluminium became apparent [18,19] leading to the use of Ca-Salts and non-metallic phosphate binders afterwards. Currently available phosphate binders include calcium-containing phosphate binders, such as calcium acetate (PhosLo^®^) and calcium carbonate. They are effective but contribute to hypercalcemia and vascular calcification [20,21,22].

Available calcium-free phosphate binders include magnesium carbonate (MagneBind^®^) and lanthanum carbonate (Fosrenol^®^) [23]. Although there were concerns of a theoretical risk of lanthanum accumulation in organs like liver, kidney and bone [24], the long-term safety of lanthanum carbonate as a phosphate binder in end-stage renal disease patients is well demonstrated [25]. Indeed, the follow-up of end-stage renal disease patients treated with lanthanum carbonate for up to six years showed that lanthanum levels remain low and that the drug only rarely causes bone and liver/biliary system-related adverse events [26]. Finally, sevelamer hydrochloride (Renagel^®^) is a polymer of cross-linked allylamine hydrochloride and epichlorohydrin. It is an anion exchange resin that releases chloride in exchange for phosphate and other anions [27,28,29]. However, sevelamer hydrochloride binds bile acids at the expense of phosphate, making it only effective in the higher pH environment of the small intestine [30,31].

The mechanism for all these types of binders is the same whereby the binder dissociates into its (metallic) cation and its corresponding anion. The cation is then free to bind dietary phosphate in the GI tract and easily excretable insoluble complexes are formed [32]. Evidence has suggested that these commonly used phosphate binders have high affinity for oxalate as well. They might therefore be used to reduce intestinal oxalate absorption in patients with hyperoxaluria while avoiding increased calcium load. Furthermore, non-calcium-based phosphate binders are associated with a decreased risk of all-cause mortality compared to calcium-based phosphate binders in patients with chronic kidney disease [33].

In quantum chemistry, density functional theory (DFT) is the most used approach to perform precise calculation of energies, charge distributions and other properties of systems relevant in a wide array of fields among which medicine and pharmaceutics [34,35,36,37]. The method allows the development of efficient models at the molecular level for real-world problems that are hard or take long to investigate experimentally. Compared to other computational tools, it offers a very good balance between chemical accuracy and computational feasibility. When employed well, the used models can be used to explain certain properties of the system at the molecular level and/ or to predict potential new materials for a specific application of interest.

In this work, DFT calculations will be used to study the binding affinity of a series of cations (i.e., Li^+^, Na^+^, Mg^2+^, Ca^2+^, Fe^2+^, Cu^2+^, Zn^2+^, Al^3+^, Fe^3+^ and La^3+^) to phosphate and oxalate in comparison to possible administration complexes such as acetate, carbonate, chloride, citrate, formate, hydroxide and sulphate. The theoretical predictions will suggest new complexes for further investigation in silico, in vitro and/or in vivo as their stability might limit the gastrointestinal absorption of oxalate and phosphate. The goal is to identify complexes that reduce urinary oxalate excretion as a treatment for hyperoxaluria and reduce serum phosphate levels as a treatment for hyperphosphatemia in chronic kidney disease, respectively. 

## 2. Computational Methods

**Figure 1:**
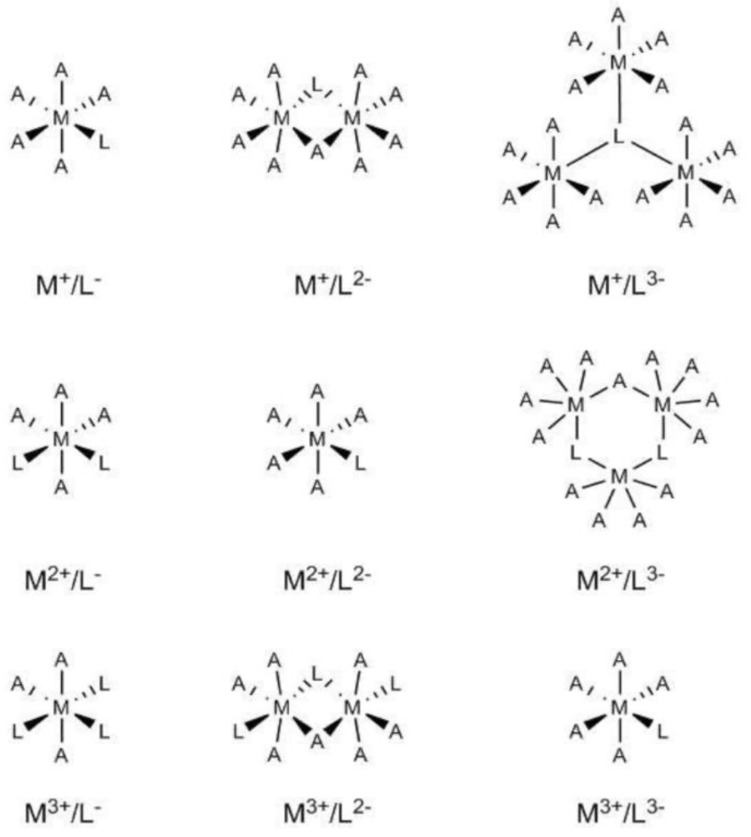
Schematic representation of the structures employed as initial geometries for the different M*_x_*L*_y_*·*n*H_2_O systems. The capital A represents the explicit water molecules added to complete the coordination sphere of the metal centres.

Initial models for the different M*_x_*L*_y_*·*n*H_2_O systems were built by considering an octahedral coordination (see Figure 1) whereby the coordination sphere of the metal centre was completed by water molecules. For some sufficiently flexible bi-dentate ligands, the number of explicit water molecules was accordingly reduced, for instance, CaC_2_O_4_·4H_2_O was employed instead of CaC_2_O_4_·5H_2_O.

It is important to point out that the quantum chemical description of such systems by means of DFT methods represents a challenging task due to the large number of degrees of freedom and the associated complexity of the potential energy surface (i.e., flat surface with the presence of a number of local minima). The following calculation protocol was used for an accurate computational description of these systems: (i) first, a preliminary geometry optimization is performed at the LC-BLYP/6-31G level of theory with relatively small step (i.e., MAXSTEP = 15) as implemented in the gaussian16 suit of programs [38], (ii) regardless of whether an equilibrium geometry is obtained or not, a subsequent geometry optimization is restarted at the same level of theory in order to compute well-converged equilibrium geometries, and (iii) a final single-point energy calculation is carried out by increasing the size of the basis set to the LC-BLYP/6-311++G(d,p) level. For the systems containing the La^3+^ cation, the LANL2TZ(f) + ECP basis set was employed [39]. It is worth mentioning that the use of the LC-BLYP functional is justified based on a recent study reported by Truhlar and co-authors [40], who have assessed the performance of different functional families in describing dissociation energies of ionic-bound systems. 

The use of the relatively small split-valence 6-311++G(d,p) basis set together with the LC-BLYP functional was determined to represent a good compromise between reliability and computational cost. In particular when taking into account that the present study focuses on trends regarding the relative stability of the complexes rather than computing accurate dissociation energies. In this respect, representative members of the different groups of systems containing a mono-, di-, and tri-valent cation with the lowest count of electrons (i.e., Li^+^, Mg^2+^, and Al^3+^ complexes) were also described with the larger basis set cc-pVTZ as an additional validation of our adopted method. In all cases, the results computed with the two employed basis sets were determined to correlate very well, being characterized by R^2^ values within the 0.96–0.99 range. The gaussian input files of the LC-BLYP/6-311++G(d,p)//LC-BLYP/6-31G single point calculations corresponding to all studied systems are available as Supplementary Materials.

In all calculations of the protocol (i) to (iii), solvent effects were considered through the Solvation Model based on Density (SMD) [41] with a dielectric constant of ε = 78.3553 corresponding to an aqueous phase. The use of implicit solvent during the preliminary optimization was found necessary to mitigate the presence of too strong negative charges in some ligands resulting in undesirable deprotonation of the explicit water molecules. For systems including cations with electron multiplicity higher than 1 (i.e., Cu^2+^ and Fe^3+^ within an *O_h_* geometry), the lowest spin states were considered. 

Complexation energies were calculated by means of the following expression:

(1) $$\Delta E=E_{M_{x}L_{y}\cdot nH_{2}O}-xE_{M}-yE_{L}-nE_{H_{2}O}$$

where $E_{M_{x}L_{y}\cdot nH_{2}O}$, $E_{M}$,$\;E_{L}$, and $E_{H_{2}O}$ are the energies (including implicit solvent effects) of the complex, the metal cation, the ligand, and the explicit solvent molecules, respectively. Basis set superposition errors (BSSE) were not considered in the present study due to the computational cost and, as mentioned before, the focus on the relative stabilities of the complexes, which is not expected to be greatly affected by these errors.

## 3. Results

In order to identify efficient phosphate (PO_4_^3−^) and oxalate (^−^OOC-COO^−^) binders, their respective complexes with various mono-, di-, and trivalent metal cations were considered in the present computational study, namely (in order of increasing charge): Li^+^, Na^+^, Mg^2+^, Ca^2+^, Fe^2+^, Cu^2+^, Zn^2+^, Al^3+^, Fe^3+^ and La^3+^. Furthermore, other common ligands present in the body such as acetate (CH_3_COO^−^), carbonate (CO_3_^2−^), chloride (Cl^−^), citrate (C_6_H_5_O_7_^3−^), formate (HCOO^−^), hydroxide (HO^−^) and sulphate (SO_4_^2−^) were also included in the work to allow comparison of the stability of the ligands of interest (phosphate and oxalate) with competing residues.

The formation energies computed through Equation (1) for all computed complexes are summarized in Table 1 whereby it has to be noted that the equilibrium geometry of the LaCl_3_·3H_2_O could not be determined despite several attempts. It is tempting to look at the columns containing the oxalate and phosphate complexation energies and perceive the lowest one as best candidate for binding the respective anions. However, as the cationic species needs to be administered in the form of a complex with a suitable ligand and all the considered ligands are already present in the body, the difference in complexation energy between a safely applicable ligand and the phosphate or oxalate ligand is of larger importance. Ideally, a stable complex of cation-ligand is replaced for a (much) more stable cation-phosphate/oxalate complex, leaving a non-toxic free ligand in the body. Therefore, it is more informative to look at the table by cationic species to assess the differences between the complexation energies with the different ligands. For convenience, more detailed, graphical representations are given below as they are discussed. 

It should be noted that the interpretation of the complexation energies should be done keeping in mind that kinetic and concentration effects might influence the formation of complexes in ways that was not included in this study. Moreover, one should take into consideration that the pH-dependency of the solubility for the different salts has not been included in the model calculations. Despite these shortcomings, the enthalpic results from the calculations can give important insights on the stability of the investigated complexes and suggest possible candidates for subsequent investigation in silico, in vitro and/or in vivo.

**Figure 2:**
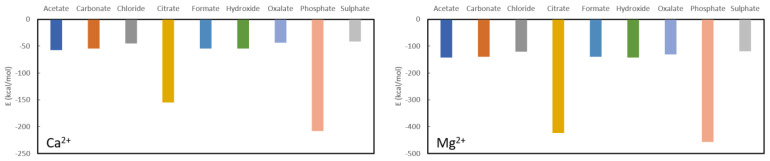
Formation energies for the complexes with Ca^2+^ (**left**) and Mg^2+^ (**right**).

From Figure 2 one can confirm the effectiveness of long-known phosphate binders, such as calcium acetate and calcium carbonate. Their complexation energies of −57.20 kcal/mol and −54.40 kcal/mol, respectively, are much smaller than the complexation energy of calcium phosphate (−208.10 kcal/mol) and a swift recomplexation of the Ca^2+^ ion with the phosphate anion is thus expected thermodynamically. From the remaining investigated ligands, which are not used as phosphate binders, formate (−54.60 kcal/mol) and hydroxide (−54.50 kcal/mol) are in the same range as acetate and carbonate. Chloride (−45.00 kcal/mol), oxalate (−43.70 kcal/mol) and sulphate (−41.50 kcal/mol) are somewhat lower in complexation energy, while citrate forms quite a stable complex with a formation energy of −155.00 kcal/mol. With that, it is also immediately seen that calcium is not a suitable chelator for oxalate as it has one of the lowest complexation energies. The latter is not in line with what is generally performed in KS prevention, namely increasing oral calcium intake through meals or administering calcium supplements to reduce oxalate excretion [42]. Probably effects are at play that are not accounted for in these model calculations, such as pH, solvent effects and/or kinetic effects.

A similar picture is seen for complexation of the investigated ligands with magnesium as shown further in Figure 2. The phosphate complexation energy with Mg^2+^ is the largest with a complexation energy of −465.80 kcal/mol. Citrate is, again, the only one that comes close with an energy of −423.30 kcal/mol, followed in descending order by acetate (−143.10 kcal/mol), hydroxide (142.10 kcal/mol), formate (−140.10 kcal/mol), carbonate (−139.60 kcal/mol), oxalate (−130.80 kcal/mol), chloride (−123.20 kcal/mol) and sulphate (−118.50 kcal/mol). It should be noted that, also here, the commonly used magnesium carbonate (often in combination with calcium acetate) is thermodynamically confirmed to be a very good phosphate binder. In fact, of the considered choices, only citrate would be a bad choice for a ligand as the difference in formation energy is not very large in comparison to phosphate. Similarly, to Ca^2+^, Mg^2+^ is expected not to be a good oxalate binder as it only complexes slightly easier with chloride and sulphate. 

**Figure 3:**
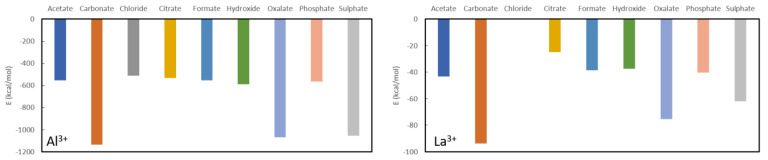
Formation energies for the complexes with Al^3+^ (**left**) and La^3+^ (**right**).

In general, aluminium (see Figure 3) appears to be the best complexing agent, by far, for all studied ligands with formation energies between −500 kcal/mol and −1200 kcal/mol. Although it has been used in the past in the form of aluminium hydroxide (Al(OH)_2_) [43], its use is no longer recommended as it is associated with aluminium toxicity in chronic kidney disease patients, characterized by vitamin D-resistant osteomalacia, microcytic anemia, bone and muscle pain, hypercalcemia and dementia [44]. Aside from these obvious limitations, its use as a phosphate binder is expected to be rather limited according to current calculations as the aluminium complexation energy (−563.6 kcal/mol) is only slightly lower than the value for aluminium acetate (−554.2 kcal/mol), aluminium chloride (−512.6 kcal/mol), aluminium citrate (−554.5 kcal/mol), aluminium formate (−554.5 kcal/mol). Moreover, the aluminium hydroxide, that was used in the past, is predicted to be a slightly more stable complex than the target aluminium phosphate complex with a formation energy of −554.5 kcal/mol. On the other hand, it is expected to be a very good oxalate binder as the corresponding complex is almost twice as stable (−1068.2 kcal/mol) as the values for the other ligands, except for sulphate which is only slightly less stable (−1055.6 kcal/mol) and carbonate which is slightly more stable (−1136.9 kcal/mol). Unfortunately, the toxic nature of aluminium remains regardless of whether it is used as a phosphate or an oxalate binder.

For lanthanum, the picture is qualitatively very similar, although all formed complexes are much less stable than for aluminium (see Figure 3). Surprisingly, the commonly used phosphate binder lanthanum carbonate is almost twice as stable as lanthanum phosphate, −93.9 kcal/mol vs. −40.3 kcal/mol. The other considered complexes are slightly less stable in the case of citrate (−24.7 kcal/mol), hydroxide (−37.2 kcal/mol), while oxalate (−75.4 kcal/mol) is almost twice as stable. Finally, complexation with sulphate is lies intermediate to these values (−61.9 kcal/mol). From these results, one can infer that, with an appropriate ligand choice, La^3+^, might serve well as an oxalate binder, much better than it is a phosphate binder.

**Figure 4:**
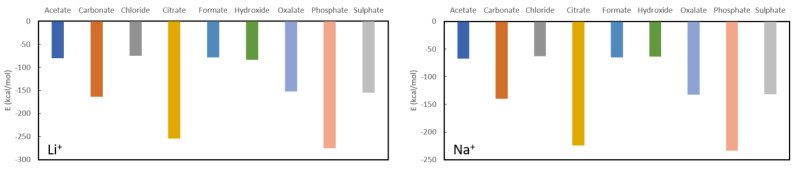
Formation energies for the complexes with Li^+^ (**left**) and Na^+^ (**right**).

After discussion of the known phosphate binders, the focus will now be on possible new candidates for phosphate/oxalate complexation. In Figure 4, the complexation energies for Li^+^ and Na^+^ are shown whereby it is seen that the predicted complexation energies are very similar (not surprising given their related positions in the periodic table). It should be noted though that the complexes formed by lithium are between 15 kcal/mol and 40 kcal/mol more stable than for sodium. Both lithium and sodium are expected to be good phosphate binders (complexation energies of −274.8 kcal/mol and −233.8 kcal/mol) and reasonable oxalate binders (−152.5 kcal/mol and −132.6 kcal/mol) when appropriate ligands are used for administration. More specifically, the use of citrate (−254.3 kcal/mol and −224.3 kcal/mol) is to be avoided for phosphate binding, while citrate, sulphate (−155.2 kcal/mol and −131.7 kcal/mol) and carbonate (−163.5 kcal/mol and −139.8 kcal/mol) are to be avoided for oxalate binding. The remaining ligands have complexation energies with respectively lithium and sodium of −80.7 kcal/mol and −66.9 kcal/mol for acetate, −75.0 kcal/mol and −62.7 kcal/mol for chloride, −79.0 kcal/mol and −64.9 kcal/mol for formate and −84.2 kcal/mol and −63.8 kcal/mol for hydroxide.

**Figure 5:**
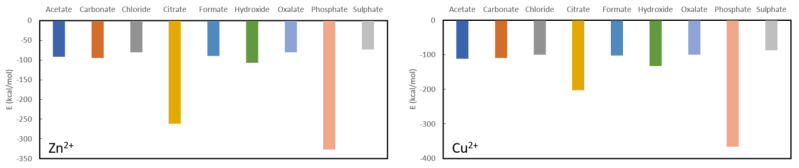
Formation energies for the complexes with Zn^2+^ (**left**) and Cu^2+^ (**right**).

The results for Zn^2+^ and Cu^2+^ are rather similar (Figure 5), although the latter binds overall about 10 kcal/mol to 30 kcal/mol more stable to the different anions. Both are predicted to be very stable in complexation with phosphate, i.e., −326.4 kcal/mol for Zn^2+^ and −365.6 kcal/mol for Cu^2+^. The latter is expected to be a better phosphate binder as, aside from the more stable absolute complexation energy, the difference with the second most stable ligand (citrate) is much larger than for zinc. Indeed, the complexation energy for zinc citrate is −261.6 kcal/mol, while for cupper citrate it is −202.9 kcal/mol. Furthermore, the remaining complexation energies for zinc are −106.8 kcal/mol for hydroxide, −94.4 kcal/mol for carbonate, −89.8 kcal/mol for formate, −80.6 kcal/mol for oxalate, −80.0 kcal/mol for chloride and −73.0 kcal/mol for sulphate, while for cupper they are −132.3 kcal/mol for hydroxide, −108.8 kcal/mol for carbonate, −102.2 kcal/mol for citrate, −100.4 kcal/mol for chloride, −99.4 kcal/mol for oxalate and −86.6 kcal/mol for sulphate. From these results, it is clear that neither cupper nor zinc are expected to be good oxalate binders.

**Figure 6:**
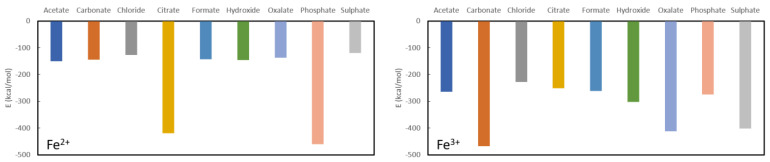
Formation energies for the complexes with Fe^2+^ (**left**) and Fe^3+^ (**right**).

Results for the final investigated cations are collected in Figure 6. It is immediately clear that Fe^2+^ may be expected to serve well as a phosphate binder with a complexation energy to phosphate of −460.3 kcal/mol in comparison to relatively low formation energies of the other studied complexes with the exception of citrate (−419.9 kcal/mol). Indeed, the remaining ligands have complexation energies of −150.1 kcal/mol for acetate, −145.1 kcal/mol for carbonate, −127.3 kcal/mol for chloride, −143.0 kcal/mol for formate, −145.4 kcal/mol for hydroxide, −137.3 kcal/mol for oxalate and −119.5 kcal/mol for sulphate. Given these numbers, Fe^2+^ cannot be expected to serve as a good oxalate binder as the complexation energy with oxalate is in the same range as or higher than the remaining investigated complexes. 

For Fe^3+^, the picture is quite different (see Figure 5) as there is a remarkably strong complexation with sulphate (−402.0 kcal/mol). No good performance as a phosphate binder is expected as the complexation energy with phosphate (−275.2 kcal/mol) is lower or of the same order as the remaining ligands: −467.2 kcal/mol for carbonate, −227.2 kcal/mol for chloride, −251.7 kcal/mol for citrate, −261.3 kcal/mol for formate and −302.0 kcal/mol for hydroxide. It is interesting to note that there is an iron-based compound that is used as a phosphate binder (Velphoro^®^, sucroferric oxyhydroxide) whereby the iron is in a 3+ state which is contradicting the present results. However, it is known that Fe^3+^ in the intestine is very rapidly reduced to Fe^2+^ for which good phosphate binding behaviour is expected. Similarly, these results also justify the use of ferric citrate (which is used in combination with calcium acetate) as phosphate binder, although the results suggest that administration in combination with another ligand, might be more suitable. Given that the complexation energy of Fe^3+^ with oxalate is only slightly higher than some of the other ligands and the conversion of Fe^3+^ to Fe^2+^ in the intestine, this cation is also not expected to show good oxalate binding behaviour.

## 4. Discussion

The present calculations confirm the suitability of the widely used calcium and magnesium phosphate binders. On the other hand, for aluminium and lanthanum, no good behaviour is expected for phosphate binding, whereby it should again be noted that several aspects from the processes at play were not included in this study (e.g., kinetic effects). Aside from these known binders, other possible candidates were identified such as Na^+^, Li^+^, Zn^2+^, Cu^2+^ and Fe^2+^. In general, it is clear that phosphate readily binds with multiple cations explaining the widespread use of these complexing agents. In absolute terms, the strongest phosphate complexes are formed with (Al^3+^ >) Fe^2+^ > Mg^2+^ > Cu^2+^ > Zn^2+^, whereby it should be noted that Al^3+^ binds much stronger with oxalate, hence the brackets.

For oxalate, the picture is very different, and it is much harder to find well-complexing cations. Indeed, we have only been able to identify aluminium as a very strong candidate for oxalate complexation, while also lanthanum [45] and Fe^3+^ show promise. Lithium and sodium also show some promise, but to a smaller extent. Interestingly, in absolute terms, the strongest complexes are formed with Al^3+^ and Fe^2+^, just as for phosphate. Relatively speaking, the most favourable cations for oxalate complexation appear to be Al^3+^ > La^3+^ > Fe^3+^ >> Li^+^ > Na^+^, with the note that only for the trivalent cations (including La^3+^) a more stable complex is formed with oxalate in comparison to phosphate. Finally, we would like to note that this result may also be relevant for the suggested capability of oxalate to lower the absorption of Fe^3+^ obtained from vegetables.

Overall, it is seen that mono- and divalent charged cations prefer complexation with citrate and phosphate, whereas trivalent cations prefer complexation with carbonate, oxalate and sulphate. As Fe^3+^ is very rapidly converted to Fe^2+^, it is probably not well-suited as an oxalate binder, as discussed previously. However, Al^3+^ and La^3+^ might stay longer in a 3+ state in the body and therefore be more efficient oxalate binders. Obviously, these kinds of effects are not accounted for in the current model as the chelator is assumed to remain in its cationic state until binding to the ligand. In vitro and/or in vivo testing is needed in order to shed light on these aspects of the process. In vitro, Lanthanum carbonate has a high affinity for oxalate across the entire pH range encountered in the gastrointestinal tract [46]. In vivo, Lanthanum carbonate reduces serum oxalate concentration and urinary Ox excretion in a rat model of hyperoxaluria induced by ethylene glycol intoxication. Additionally, Lanthanum carbonate do not affects calciuria in rats with normal renal function and in healthy volunteers [47]. Importantly, citrate (current kidney stones recurrence preventive therapy) does not have any effect on lanthanum absorption. The bioavailability of La is very low (0.00127 ± 0.0008%) and the urinary excretion is less than 1% because >99% of La is excreted by the bile, which is the main reason for use in patients with ESRD at anuric stage [25].

More general, it may be inferred that trivalent cationic species that remain relatively long in a 3+ state in the intestine are worth investigating as oxalate binders, while cations in a 2+ state are suitable candidates for phosphate binding.

In previous studies, a clear trend has been identified between the ionic radius and complexation energy for the mono- and divalent cations, which was reproduced here for citrate and phosphate [48]. For the trivalent cations the relation with the ionic radius is less clear. Furthermore, in agreement with the laws of electrostatics, the stability of the complexes increases as the cation charge increases.

Based these trends, a further prediction can be made for other possible oxalate binders to be studied theoretically and experimentally. The potential candidate should have a 3+ charge and a large ionic radius of about 100 pm and not be toxic. Based on this, neodymium could be proposed as a candidate for further investigation as a possible replacement for lanthanum [25]. It has already been used in pharmaceutical applications in gynaecology in the early 1900s and as dopants for laser technology, while being cheaper (in 2020) than lanthanum. However, it should be said that the clinical safety of orally administered neodymium is unknown to date and deserves preclinical studies. Studies on the possible applications of neodymium in this context are underway.

Finally, some of the shortcomings of this work need to be addressed, which are mostly related to the fact that this is computational work including only static DFT calculations. This obviously means that no kinetic effects are taken into account as has been highlighted throughout the work already. For computational reasons, it was in this work assumed that all the complexes have octahedral coordination, which may not be ideal for all complexes considered. In fact, this may be the cause of not finding an equilibrium geometry for the LaCl_3_·2H_2_O complex. Another issue related to computational complexity is the fact that the considered complexes may actually form larger clusters composed of more than one metal ion. Obviously, doing such large calculations is limited by computational power at this level of theory. Furthermore, no temperature effects were taken into account as DFT calculations assume a temperature of 0 K, which is of course far from physiological temperature. An attempt was made to correct the formation energies by calculation Gibbs formation energies at 300 K as can be seen in the Supplementary Materials. As this correction seemed to not immediately resolve some of the computational inaccuracies, a further correction of these results was considered outside the scope of this work. Despite these inherent limitations, the authors believe that important insights on these systems were acquired and are promising enough to pursue through more refined calculations as well as—more importantly—experimental work.

## 5. Conclusions

Using density functional theory, a series of nine anionic ligands was assessed on their affinity to form a complex with a series of 10 different cations in octahedral coordination in aqueous medium. From this systematic study the complexation energy of some known complexes used as phosphate binders (calcium acetate, magnesium carbonate, aluminium salts, …) were confirmed and quantified. Independently, the results from US Patent 2002/0155168 A1 were recovered, providing a fundamental explanation to their findings [49]. Furthermore, a series of complexes was identified that could serve as phosphate and/or oxalate binders and is worthy of further in silico, in vitro and/or in vivo investigation. In general, it was found that divalent cations (Fe^2+^, Ca^2+^, Mg^2+^ and Zn^2+^) are most suitable for complexation with phosphate, while trivalent cations (Fe^3+^, Al^3+^ and La^3+^) show more affinity for oxalate. Finally, based on the observed trends, we propose neodymium as another interesting cation to be investigated for oxalate binding.

## Figures and Tables

**Table 1:** Formation energies for all complexes considered in the present study in kcal/mol. For lanthanum chloride, no stable complex was found.

	Acetate	Carbonate	Chloride	Citrate	Formate	Hydroxide	Oxalate	Phosphate	Sulphate
Li^+^	−80.7	−163.5	−75.0	−254.3	−79.0	−84.2	−152.5	−274.8	−155.2
Na^+^	−66.9	−139.8	−62.7	−224.3	−64.9	−63.8	−132.6	−233.8	−131.7
Mg^2+^	−143.1	−139.6	−121.2	−423.3	−140.1	−142.1	−130.8	−456.8	−118.5
Ca^2+^	−57.2	−54.4	−45.0	−155.0	−54.6	−54.5	−43.7	−208.1	−41.5
Fe^2+^	−150.1	−145.1	−127.3	−419.9	−143.0	−145.4	−137.3	−460.3	−119.5
Cu^2+^	−111.4	−108.9	−100.4	−202.9	−102.2	−132.3	−99.4	−365.6	−86.6
Zn^2+^	−91.4	−94.4	−80.0	−261.6	−89.8	−106.8	−80.6	−326.4	−73.0
Al^3+^	−554.2	−1136.9	−512.6	−534.2	−554.5	−590.2	−1068.2	−563.6	−1055.6
Fe^3+^	−264.3	−467.2	−227.2	−251.7	−261.3	−302.0	−412.2	−275.2	−402.0
La^3+^	−43.1	−93.7	−−	−24.7	−38.5	−37.2	−75.4	−40.3	−61.9

## Data Availability

Not applicable.

## Supplementary Materials

The following are available online at https://www.mdpi.com/article/10.3390/nano11071763/s1, Table S1: Formation energies for all complexes considered in the present study.
