# Supplementary material for: In Search of an Efficient Complexing Agent for Oxalates and Phosphates: A Quantum Chemical Study
Source: Nanomaterials (Basel). 2021 Jul 6;11(7):1763. doi: 10.3390/nano11071763 (PMC8308151; doi:10.3390/nano11071763)
Supplement: Supplementary file 1 [file nanomaterials-11-01763-s001.zip › nanomaterials-1268279-supplementary.pdf]

Supplementary Materials

In Search of an Efficient Complexing Agent for Oxalates and Phosphates: A Quantum Chemical Study

Jelle Vekeman [1,*,†], Javier Torres [2,3,*], Cristina Eugenia David [4,5], Els Van de Perre [6], Karl Martin Wissing [6], Emmanuel Letavernier [7,8,9], Dominique Bazin [10,11], Michel Daudon [7,8,9], Agnieszka Pozdzik [4,5] and Frederik Tielens [1,*]

1 General Chemistry (ALGC), Materials Modelling Group, Vrije Universiteit Brussels, 1050 Brussels, Belgium
2 Grupo de Química Computacional y Teórica (QCT-USFQ), Departamento de Ingeniería Química, Universidad San Francisco de Quito (USFQ), Diego de Robles y Vía Interoceánica, Quito 17-1200-841, Ecuador
3 Instituto de Simulación Computacional (ISC-USFQ), Departamento de Ingeniería Química, Universidad San Francisco de Quito (USFQ), Diego de Robles y Vía Interoceánica, Quito 17-1200-841, Ecuador
4 Kidney Stone Clinic, Nephrology Department, Centre Hospitalier Universitaire, Brugmann Hospital, 1020 Brussels, Belgium; CristinaEugenia.DAVID@chu-brugmann.be (C.E.D.); Agnieszka.POZDZIK@chu-brugmann.be (A.P.)
5 Faculty of Medicine, Université Libre de Bruxelles (ULB), 1050 Brussels, Belgium
6 Nephrology Department, Universitair Ziekenhuis Brussel, Vrije Universiteit Brussel, 1090 Brussels, Belgium; Els.VandePerre@uzbrussel.be (E.V.d.P.); KarlMartin.Wissing@uzbrussel.be (K.M.W.)
7 Sorbonne Universités-UPMC Univ. Paris 06, UMR S 1155, 75020, Paris, France; emmanuel.letavernier@aphp.fr (E.L.); michel.daudon@aphp.fr (M.D.)
8 INSERM, UMR S 1155, 75020, Paris, France
9 Explorations Fonctionnelles Multidisciplinaires, AP-HP, Hôpital Tenon, 75020, Paris, France
10 Institut de Chimie Physique, UMR CNRS 8000, Université Paris Saclay, Bâtiment 350, CEDEX, 91405 Orsay, France; bazin@lps.u-psud.fr
11 Laboratoire de Physique des Solides, UMR CNRS 8502, Université Paris-Saclay, Bâtiment 510, CEDEX, 91405 Orsay, France
* Correspondence: jelle.vekeman@ugent.be (J.V.); jtorres@usfq.edu.ec (J.T.); frederik.tielens@vub.be (F.T.)
† Current address: Center for Molecular Modeling (CMM), Ghent University, Technologiepark-Zwijnaarde 46, 9052 Zwijnaarde, Belgium.

Table S1. Gibbs free Formation energies for all complexes considered in the present study in kcal/mol. For lanthanum chloride, no stable complex was found.

	**Acetate**	**Carbonate**	**Chloride**	**Citrate**	**Formate**	**Hydroxide**	**Oxalate**	**Phosphate**	**Sulphate**
**Li^+**	−14.3	−35.6	−13.5	−41.3	−12.4	−22.4	−26.4	−67.4	−24.7
**Na^+**	−3.5	−18.8	−2.8	−15.9	2.5	−5.4	−9.7	−27.7	−10.3
**Mg^{2+}**	−73.4	−70.8	−67.3	−223.8	−71.0	−78.6	−76.8	−248.4	−62.4
**Ca^{2+}**	7.6	9.1	8.6	28.8	10.9	2.9	6.3	−10.3	8.1
**Fe^{2+}**	−77.0	−72.9	−65.6	−215.2	−70.0	−77.9	−77.7	−242.9	−60.4
**Cu^{2+}**	−44.2	−41.2	−39.6	−5.7	−33.0	−66.2	−42.2	−159.9	−31.7
**Zn^{2+}**	−24.3	−25.1	−21.1	−67.4	−19.8	−40.3	−24.7	−121.4	−16.8
**Al^{3+}**	−479.2	−996.3	−452.9	−459.3	−480.1	−522.3	−927.4	−491.3	−909.7
**Fe^{3+}**	−186.4	−319.3	−167.7	−174.1	−184.5	−231.6	−267.9	−201.9	−255.1
**La^{3+}**	23.2	33.3		38.0	26.9	17.6	51.0	20.4	64.1

The Gibbs free formation energy was estimated by calculating the Gibbs free formation energy of the complex at the smaller basis set (see computational methods) and correcting it using the formation energies as calculated in the main text at both the large and small basis sets:

$$G_{\text{formation}} = G_{\text{small}} - E_{\text{small}} + E_{\text{large}} \quad \text{(S1)}$$
